# Supplementary material for: Transcriptome Analysis Reveals Vimentin-Induced Disruption of Cell–Cell Associations Augments Breast Cancer Cell Migration
Source: Cells. 2022 Dec 13;11(24):4035. doi: 10.3390/cells11244035 (PMC9776984; doi:10.3390/cells11244035)
Supplement: Supplementary file 1 [file cells-11-04035-s001.zip › DEGs and enrichment analysis.zip!/DEGs and enrichment analysis/WT_VIMvsVector KEGG/ALL/WT_VIMvsVector_ALL.pathway_map.zip!/WT_VIMvsVector_ALL.pathway_map/src/hsa01523.html]

hsa01523


- hsa:6573

- Up regulated genes

ENSG00000173638(1.83206395292807)

- hsa:10257

- Down regulated genes

ENSG00000125257(-3.20715697961916)

- hsa:7298

- Up regulated genes

ENSG00000176890(1.69666136230634)

- hsa:6573

- Up regulated genes

ENSG00000173638(1.83206395292807)

- hsa:6573

- Up regulated genes

ENSG00000173638(1.83206395292807)

Close
